# Mechanical Characteristics of Hardened Concrete with Different Mineral Admixtures: A Review

**DOI:** 10.1155/2014/875082

**Published:** 2014-01-29

**Authors:** Tehmina Ayub, Sadaqat Ullah Khan, Fareed Ahmed Memon

**Affiliations:** ^1^Civil Engineering Department, NED University of Engineering and Technology, Karachi 57270, Pakistan; ^2^Urban Engineering Department, NED University of Engineering and Technology, Karachi 57270, Pakistan; ^3^Civil Engineering Department, Mehran University of Engineering and Technology, Jamshoro 76062, Pakistan

## Abstract

The available literature identifies that the addition of mineral admixture as partial replacement of cement improves the microstructure of the concrete (i.e., porosity and pore size distribution) as well as increasing the mechanical characteristics such as drying shrinkage and creep, compressive strength, tensile strength, flexural strength, and modulus of elasticity; however, no single document is available in which review and comparison of the influence of the addition of these mineral admixtures on the mechanical characteristics of the hardened pozzolanic concretes are presented. In this paper, based on the reported results in the literature, mechanical characteristics of hardened concrete partially containing mineral admixtures including fly ash (FA), silica fume (SF), ground granulated blast furnace slag (GGBS), metakaolin (MK), and rice husk ash (RHA) are discussed and it is concluded that the content and particle size of mineral admixture are the parameters which significantly influence the mechanical properties of concrete. All mineral admixtures enhance the mechanical properties of concrete except FA and GGBS which do not show a significant effect on the strength of concrete at 28 days; however, gain in strength at later ages is considerable. Moreover, the comparison of the mechanical characteristics of different pozzolanic concretes suggests that RHA and SF are competitive.

## 1. Introduction

Many researchers addressed the deficiencies of concrete and some of them made significant efforts to improve the performance of concrete, especially permeability and durability of concrete as these are the immense concerns of the researchers. The existing literature related to pozzolanic concretes shows that the use of mineral admixtures reduces the porosity of concrete if cement content is partially replaced by mineral admixture; therefore, the demand of blended cement has increased globally to produce denser to impermeable concretes [1], along with improving the strength of concrete such as compressive, tensile, and flexure ones. On one side, these mineral admixtures enable concrete to exhibit greater resistance against harmful solutions (e.g., acid and chemicals, etc.), freezing and thawing, chloride ion penetration, sulphate attack and carbonation, and so forth and, on the other side, they are important contributors for sustainable environment as partial replacement of cement and often called as “less energy intensive cementitious materials” [2]. Use of mineral admixtures is such an advantage that some cement companies have started manufacturing fly ash cement. Fly ash has also been used as a partial replacement of fine aggregate and has been recommended for structural use [3].

Among several available types, the most commonly used mineral admixtures are fly ash (FA), silica fume (SF), ground granulated blast furnace slag (GGBS), metakaolin (MK), and rice husk ash (RHA). Researchers well reviewed the properties of mortar and/or concrete containing different mineral admixtures [2, 4–6]; for example, MK in the literature has been demonstrated as an effective pozzolan exhibiting greater durability and resistance against solutions from harmful wastes due to improved pore configuration [7]. Moreover, researchers also compared the properties of few mineral admixtures; for example, Mehta and Gjørv [8] compared the properties of Portland cement concrete containing condensed silica fume (SF) and fly ash (FA), Jianyong and Yan [9] and Bágel [10] compared SF and ground granulated blast furnace slag (GGBS), Justice et al. [11] and Poon et al. [12] compared SF and MK, and Nehdi et al. [13] compared SF and rice husk ash (RHA). Poon et al. [12] compared the results of high performance cement pastes containing MK with ordinary Portland cement (OPC) pastes and those containing SF and FA. Despite such a profound literature available, a combined review and comparison among pozzolanic concretes partially containing FA, SF, GGBS, MK, and RHA is missing, which is deemed needed.

## 2. Properties of Hardened Concrete

Performance of concrete is evaluated from mechanical properties which include shrinkage and creep, compressive strength, tensile strength, flexural strength, and modulus of elasticity. But compressive strength of concrete is the most important characteristic and it is generally assumed that an improvement in concrete compressive strength will improve its mechanical properties; however, in case of concrete in which cement is partially replaced by mineral admixtures, all mechanical properties are not directly associated with compressive strength and the effects of the same amount of different mineral admixtures on the mechanical properties of hardened concrete are not same. This difference of the effects of different minerals on the mechanical properties is as follows.

### 2.1. Pore Size and Porosity

**Figure 1:**
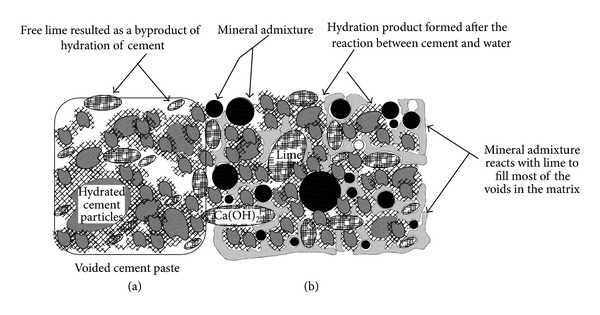
(a) Formation of lime as a byproduct of hydration of Portland cement resulting into a porous paste. (b) Pozzolanic reaction between lime and mineral admixture to fill the interstitial spaces.

Mechanical properties of concrete are closely related to its porosity and pore dispersion [14]. It is reported in the literature that the addition of mineral admixture considerably refines the pore configuration by reducing the pore size and porosity. As shown in Figure 1, after initial hydration of cement, hydrated limes (Ca(OH)_2_) form. Due to less or limited solubility, this hydrated lime remains independent in the interstitial spaces. If moisture is available, mineral admixture reacts with lime to form tricalcium silicate which refines the pore configuration of the cement matrix. It is important to mention that the rate and speed of this reaction are very much dependent on the pozzolanic nature of the mineral admixture; therefore to attain good results, silica in mineral admixture should be amorphous, glassy, or reactive. Thus, the parameters representing the pore configuration, that is, pore size and porosity, are significantly different for each partially replaced cement pastes with different mineral admixtures, even if the amount of cement replacement and water binder ratio is the constant.

In hydrated cement research, mercury intrusion porosimetry (MIP) has been used as a tool for years to quantify the distribution of pore sizes in cement pastes.  Table 1 shows significant reduction in the pore diameter with the increasing curing age showing the effectiveness of FA, MK, SF, and GGBS as partial cement replacing material. Similarly, porosity has been reduced due to decrease in pore diameter as shown in Table 2. The particle size of mineral admixtures plays an important role in final pore diameter and porosity of concrete as depicted by Chindaprasirt et al. [15]. They [15] experimentally investigated the influence of inclusion of class F FA on the porosity and pore size distribution of hardened cement pastes by replacing 0, 20, and 40% cement content and found that the total porosity and capillary pores increased as compared to the control cement pastes at all ages, but the use of finer FA (named as classified FA) at any replacement content may considerably reduce the porosity as shown in Table 2. It is interesting that FA used in the study [12] resulted into a larger pore diameter at 28 and even at 90 days also, in comparison to the FA used in the study [15], though w/b ratio was higher in the later study. From the results of study [12] and study [15], it may be inferred that FA concrete is highly sensitive to the curing procedure and curing period and this might be the reason of these contradicted results. This has been also confirmed by Ramezanianpour and Malhotra [16] who investigated the performance of SF, FA, and slag concretes under four different curing procedures and reported that, through continuous moist curing, lowest porosity can be achieved. It has been mentioned that if the duration of moist curing will be lesser, then resulted concrete will be highly porous and permeable [16]. It is also found that use of slag produces a very low permeable slag concrete, but it is more sensitive to the curing regime and slag content [16].

Poon et al. [12] presented the results of pore diameter and total porosity for MK, SF, and FA concrete (refer to Tables 1 and 2) and mentioned that improved pore configuration (i.e., lesser porosity) can be achieved by using MK in comparison to the SF. Moreover, the porosity of MK and SF pastes was found to be lesser than control at all replacement contents and at all ages. According to the results, at 20% replacement, pore diameter and porosity of MK paste were remarkably lesser than FA cement pastes, and, at 5% and 10% replacement, pore diameter and porosity of cement paste containing MK are lesser than those with SF. Similarly, Bágel [10] presented the results of SF concrete with and without slag as shown in Table 2 and it has been found that increase in SF content is not favourable for porosity and inclusion of slag is more beneficial in reduction of porosity.

Khatib and Wild [17] presented the results of pore structure of the cement pastes in which 5, 10, and 15% cement content were replaced by MK; however water binder ratio of 0.55 was kept constant. Results of pore structure of the pastes cured for 3 to 365 days were examined through mercury intrusion porosimetry (MIP). The largest pore radii have been reported as larger than 0.02 *μ*m; however, pore radii were observed to be decreased for those specimens in which MK content was higher and they were cured for the longer period. Moreover, they [17] observed a higher rate of pore refinement up to the initial curing period of 14 days and after that increase in the pore size was lesser. Kostuch et al. [18] examined the microstructure and pore size distribution of the mortar specimens cast by adding 20% MK which significantly reduced the average pore size and water absorption rate. Bredy et al. [19] also reported the same conclusion after examining the MK paste in which content of cement replacement with MK was 10 to 50%. The reported pore size is lesser than 0.03 *μ*m and the porosity of MK incorporating cement pastes was higher than OPC except for those cement pastes in which content of cement replacement was 10 and 20%.

Collins and Sanjayan [20] verified that alkali-activated slag paste (AASP) contains higher numbers of small size pores as compared to OPC paste (refer to Table 1); therefore AASP were less porous than OPC as shown in Table 2. The addition of RHA in concrete reduces the porosity of concrete; however, the interfacial zone porosity of the RHA composite was observed to be higher than that of the SF composite [21]. The reduction in pore size is due to the reaction between RHA and calcium hydroxide to form C–S–H gel [22]. El-Dakroury and Gasser [22] and Ganesan et al. [5] suggested the optimum replacement content of cement by RHA as 30% and that the use of RHA higher than 30% adversely affects the permeability and strength of concrete.

### 2.2. Drying Shrinkage and Creep

**Figure 2:**
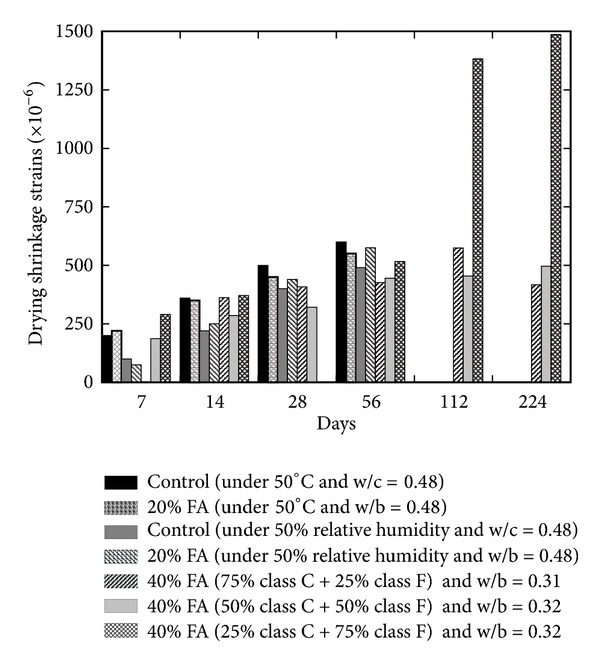
Effect of FA content and type on the drying shrinkage of FA concrete [25, 26].

**Figure 3:**
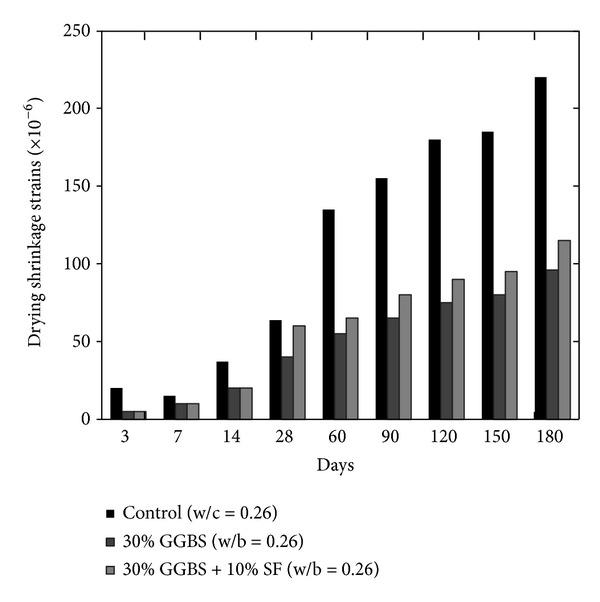
Effect of plain and blended GGBS on the drying shrinkage of GGBS concrete [9].

**Figure 4:**
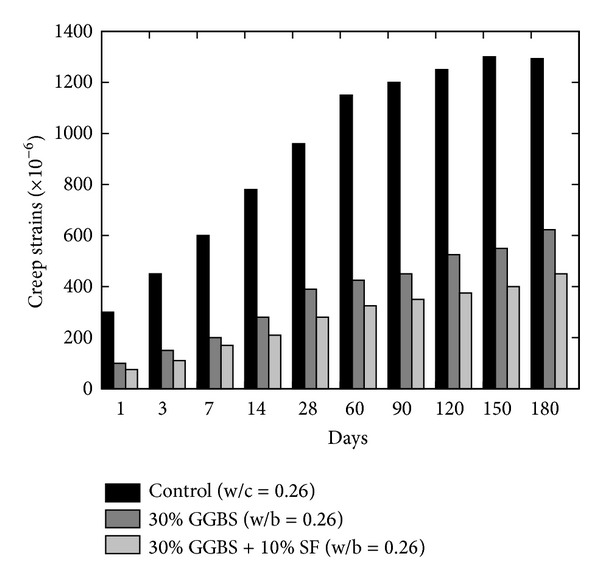
Effect of plain and blended GGBS on the creep strain of GGBS concrete [9].

Drying shrinkage property of pastes and/or concrete is usually associated with the loss of adsorbed water from the material. This property is very much significant in porous concrete, especially aerated concrete due to higher total porosity (40–80%) and specific surface of pores (around 30 m^2^/g) [14]. Decrease in the pore radii results in a higher percentage of pores and results in increased shrinkage [14]; however this property is usually related to the aggregate quality and volume; therefore, shrinkage in the paste is higher than concrete. According to Collins and Sanjayan [20], pore radius is an important parameter to determine the magnitude of shrinkage instead of the quantity of moisture loss. Higher temperature and lower humidity significantly influence shrinkage. Size of the capillary pores in the pastes and in the aggregate paste interface zone is reduced; thus capillary pressure increases as mineral admixture refines the pore configuration by reducing the pore diameter; therefore, shrinkage in concrete containing mineral admixtures will be lower due to reduced pore size as compared to OPC. This behaviour of concrete containing mineral admixtures against creep and shrinkage has been confirmed in the literature. Ghosh and Timusk [23] confirmed that good quality FA reduces the creep and drying shrinkage of concrete; however the mechanism and behaviour of FA and OPC concrete are the same [24]. Lohtia et al. [24] observed the creep behaviour of FA concrete by replacing cement up to 25% and showed that, at the higher replacement level of more than 15%, creep strains were slightly higher; therefore, 15% cement FA content is the optimum. Generally, in FA concrete, the water cement ratio is kept low because FA provides a lubricating effect which reduces water demand and consequently reduces drying shrinkage of concrete. Higher strength of FA concrete also causes smaller stress-strength ratio and ultimately lower creep than ordinary concrete. Almusallam [25] reported lower drying shrinkage in the hardened FA concrete with 20% cement content as compared to the OPC concrete when specimens were exposed to 10% humidity condition at 50°C temperature. The early age shrinkage in FA and OPC specimens was observed to be similar, but later ages (i.e., 14 to 98 days) were observed to be lower for FA concrete specimens. Similarly, the comparison of FA and OPC concrete at 50% humidity and 23°C (i.e., at room temperature) showed higher amount of shrinkage in FA concrete. Naik et al. [26] reported the results of FA concrete in which class C and class F fly ashes were used together in varying proportions. The comparison of both studies shows that the drying shrinkage of concrete increases with time with or without use of FA and water/cement ratio is a very important and significant parameter to reduce the drying shrinkage as shown in Figure 2. At fixed water/cement ratio and FA content, humidity (less than 50%) negatively influences the drying shrinkage property of FA concrete; however this trend is the opposite in OPC as shown in Figure 2. Moreover, the results reported by Naik et al. [26] show that the cement paste, in which 75% class C and 25% class F fly ashes were used, has an equivalent shrinkage to that containing 50% class C and 50% class F fly ashes. Poor resistance against shrinkage was observed in the cement paste containing 25% class C and 75% class F fly ashes [26]. On the other side, inclusion of GGBS does not significantly influence the drying shrinkage of concrete [27]. However ultrafine GGBS was examined alone and along with SF by Jianyong and Yan [9], and it was found that ultrafine GGBS caused a maximum reduction in drying shrinkage in comparison of ordinary concrete and concrete with GGBS and SF as shown in Figure 3. On the contrary, the rate of creep is lower in comparison to ordinary concrete; while the combination of GGBS and SF caused the lowest creep (refer to Figure 4). In the literature, it has been reported that, at the replacement level of more than 70 percent GGBS, the reduction in basic creep is up to 50% [27].

**Figure 5:**
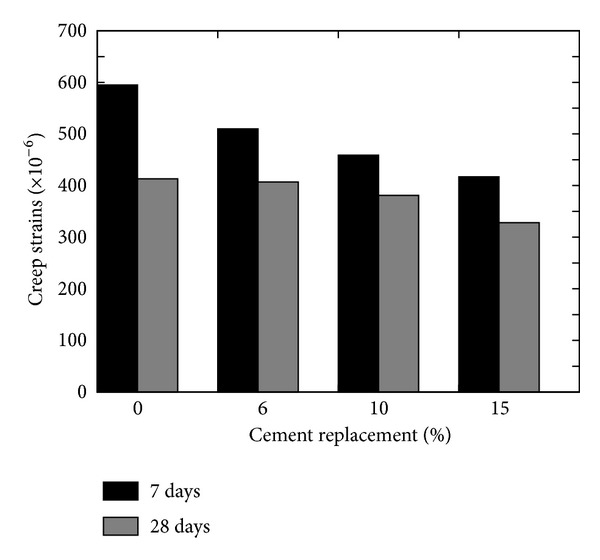
Effect of cement replacement content on the creep strain of SF concrete (at w/b = 0.35) [29].

Chung [28], after reviewing the mechanical characteristics of SF concrete, quoted that the addition of untreated and treated SF in cement pastes reduces drying shrinkage and creep rate, but the performance of treated SF is far better than plain and untreated SF concrete; however, in the literature, it is mentioned that shrinkage in cement pastes increases and the addition of the compensator is quite common [27]. Mazloom et al. [29] investigated the effect of 0, 6, 10, and 15 percent cement replacement by SF on the drying shrinkage and specific creep of high strength concrete. They reported that percentage of the replacement does not significantly influence the total shrinkage, but the autogenous shrinkage increases and drying shrinkage decreases with the percent increase in the replacement content (refer to Figure 5). Moreover, they [29] reported an equivalent rate of decrease in total and basic creep of SF concrete with the increase in the SF replacement level and reported negligible change in the drying creep.

**Figure 6:**
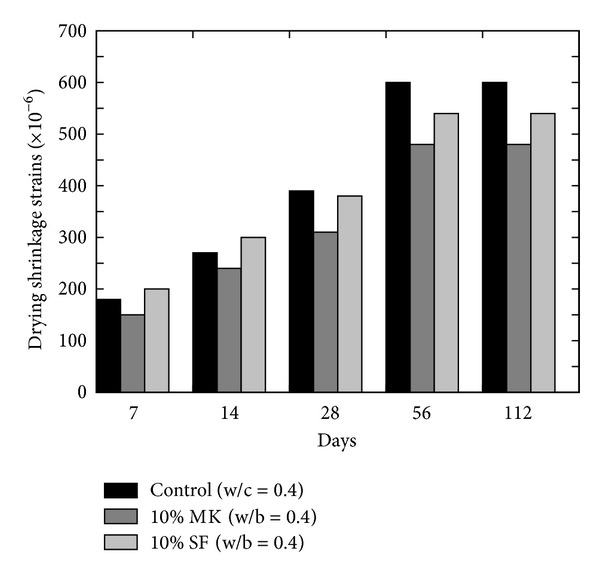
Comparison of MK and SF concretes against drying shrinkage [30].

The inclusion of MK reduces the porosity and permeability of the concrete and consequently reduces the drying shrinkage. Zhang and Malhotra [30] through experimental investigation proved that 10% replaced MK concrete had lowered the drying shrinkage than SF concrete and ordinary Portland concrete as shown in Figure 6. Brooks and Johari [31], through experimental investigation on the concretes in which content of cement replacement was up to 15% with 5% variation, showed that use of MK is advantageous in terms of reducing young age autogenous shrinkage, particularly at higher contents of cement replacement; however, at later ages, it is increased. The amount of total autogenous shrinkage increased at 5% and it is decreased at 10 and 15% of MK content. Wild et al. [32] replaced cement by 0, 5, 10, 15, 20, and 25% of MK to investigate the chemical shrinkage and autogenous shrinkage. According to their results, chemical shrinkage increased in the specimens containing 0 and 15% MK; however, above 15% MK content, chemical shrinkage decreased. On the other hand, to reduce the autogenous shrinkage, the amount of MK replacement varied between 0 and 10 percent and, beyond 15% replacement, autogenous shrinkage increased.

**Figure 7:**
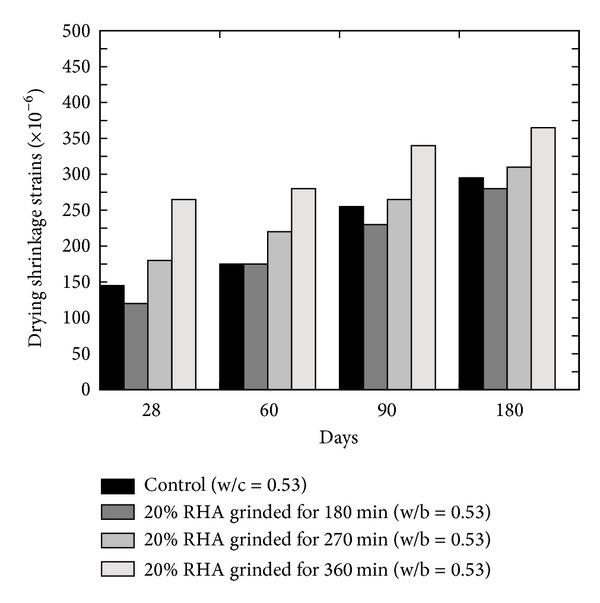
Effect of RHA fineness on the drying shrinkage of RHA concrete [33].

Habeeb and Fayyadh [33] proposed recommendation after using three different average particle sizes of RHA and with 20% RHA content as shown in Figure 7 that drying shrinkage of RHA concrete depends on the average particle size; that is, finer RHA reduces the amount of drying shrinkage. According to Zhang and Malhotra [34], the drying shrinkage of high strength concrete without cement replacement and those incorporating up to 10% SF and 10% RHA is comparable.

### 2.3. Compressive Strength

**Figure 8:**
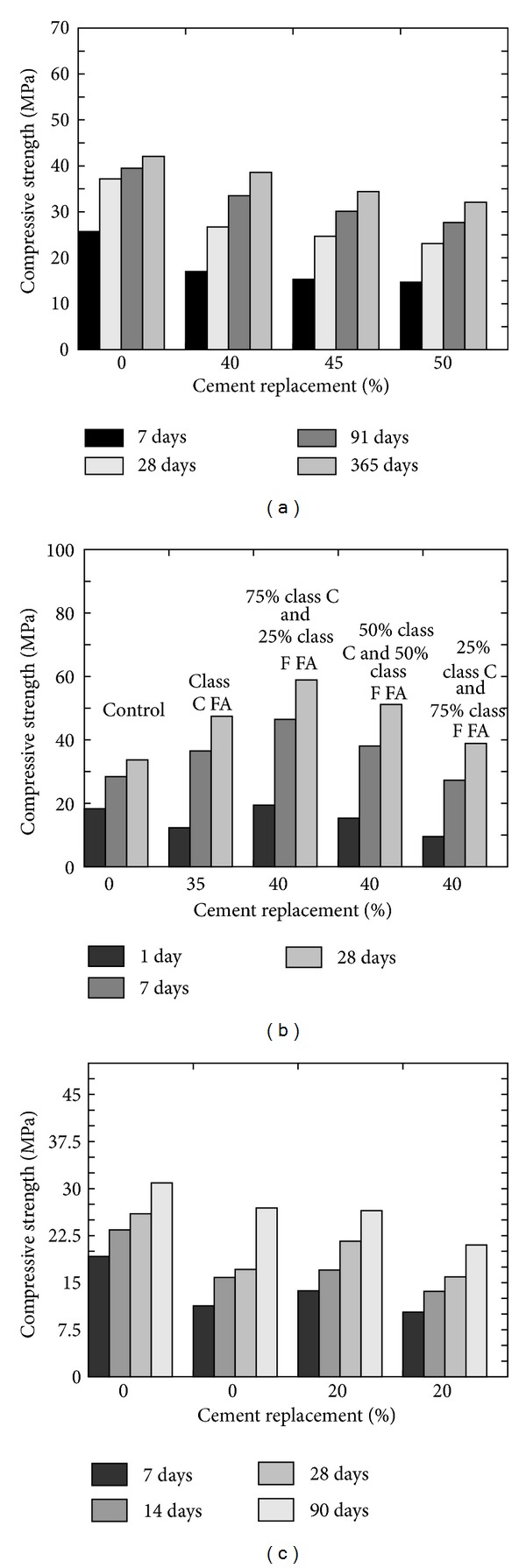
(a) Effect of cement replacement (w/b ≈ 0.4) [36], (b) FA types [26], and (c) water binder ratio (w/b = 0.48 and 0.5) [25] on the compressive strength of FA concrete.

Compressive strength of concrete is an indexing property as concrete is designed to carry compressive loadings. Therefore, determining this important property is the foremost priority while dealing with any type of concrete. Almusallam [25] determined all mechanical characteristics of hardened FA concrete by replacing 20% cement content with FA and concluded that inclusion of FA results in higher compressive strength on later ages (refer to Figure 8). The slow reactivity and lesser surface area of the FA are the reason of slower compressive strength gain; therefore Mehta and Gjørv [8] recommended the combined use of normal and highly active mineral admixtures. This recommendation was based on the 7 and 28 days of compressive strength results of concrete in which 30% cement content was replaced with FA. They [8] found a decrease in the compressive strength as compared to the normal concrete; however 28-day strength was found to be higher when mixtures of FA and SF were used as 50 : 50. Pala et al. [35] also confirmed the decrease in the early compressive strength and increase in long-term compressive strength of fly ash concrete. Naik et al. [26] evaluated the effects on the compressive strength by using mixed ASTM class C and class F FA on the mechanical and durability related properties of concrete (refer to Figure 8). Three different mix proportions of ASTM class C and class F FA were used; however, the overall content of cementitious materials in all three mixes was the same as 40%. They [26] reported that performance of mixed ASTM class C and class F FA concrete is comparatively better than the concrete without FA or only containing ASTM class C FA. In another study conducted by Siddique [36], it is shown that, at a higher cement replacement level with FA, compressive strength is reduced (refer to Figure 8).

**Figure 9:**
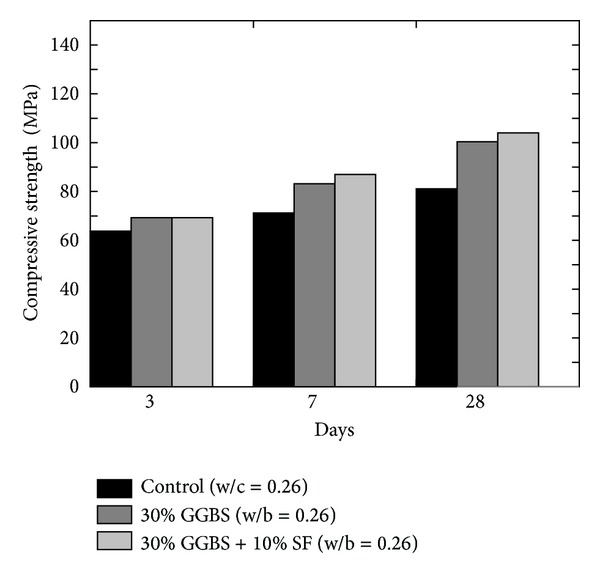
Effect of plain and blended GGBS on the compressive strength of GGBS concrete [9].

Similar to FA concrete, early strength gain with GGBS is slower than the ordinary Portland cement concrete possibly due to slow hydration process and higher amount of slag content, but long-term strength is higher than ordinary concrete if moisture remains available for further reaction between primary hydration product Ca(OH)_2_ and GGBS [9, 27]. Jianyong and Yan [9] compared the results of plain concrete, GGBS concrete, and blended concrete incorporating GGBS and SF as shown in Figure 9. According to their results, highest compressive strength was achieved with blended concrete.

In pure cement replaced concrete, high compressive strength is generally the first property which is related to the SF concrete. It has been reported in the literature that the compressive strength of concrete increases if concrete contains SF between 30 and 100 [27]. SF concrete has been used in many important building structures such as PETRONAS Tower (Malaysia), which has been constructed with the concrete having compressive strength of 100 MPa [27]. Similar results are quoted by Pala et al. [35] who described that, at any content of cement replacement by SF, the early compressive strength of concrete increases; however, compressive strength decreases at later ages. They [35] also compared the compressive strength of SF concrete to FA concrete and demonstrated that addition of SF produces highest increase in the early compressive strength in comparison with all concrete containing different amounts of FA content due to higher pozzolanic nature. Bágel [10] showed that there is an inverse relationship between compressive strength and SF content in blended mortars. Moreover, for fixed w/c ratio, addition of SF increases the compressive strength.

**Figure 10:**
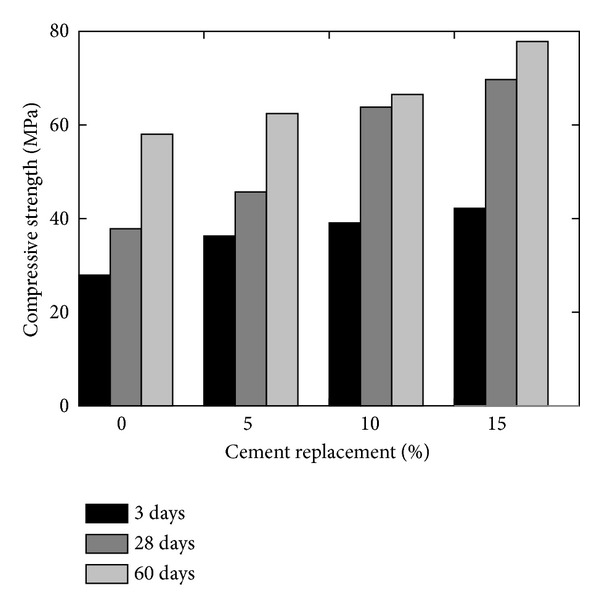
Effect of cement replacement on the compressive strength of MK concrete (w/b = 0.38) [38].

Effect of incorporating the GGBS and MK on the strength of concrete is investigated by Khatib and Hibbert [37] who partially replaced Portland cement with GGBS up to 80% and MK up to 20% keeping the constant w/b ratio of 0.5 for all mixes and concluded that the incorporation of MK increases the strength due to fast reactive nature, especially during the early ages of curing, and inclusion of GGBS reduces the strength of concrete during the first 28 days as GGBS reacts slowly at young age; however, the strength of concrete in which cement was replaced with GGBS up to 60% was observed to be increased after 28 days as shown in Figure 9. Qian and Li [38] mentioned that the younger and older age compressive strength of MK concrete substantially increased with the increase in MK content that is at 0, 5, 10, and 15% as shown in Figure 10. They [38] quoted an increase of 51% in compressive strength when cement content was replaced by 15% MK and specimens were cured for 3 days. At 3 days, the compressive strength of the specimens having 10% and 15% MK is found to be higher than 28 days compressive strength of OPC concrete. Thus, the addition of MK has a prominent influence on early strength.

**Figure 11:**
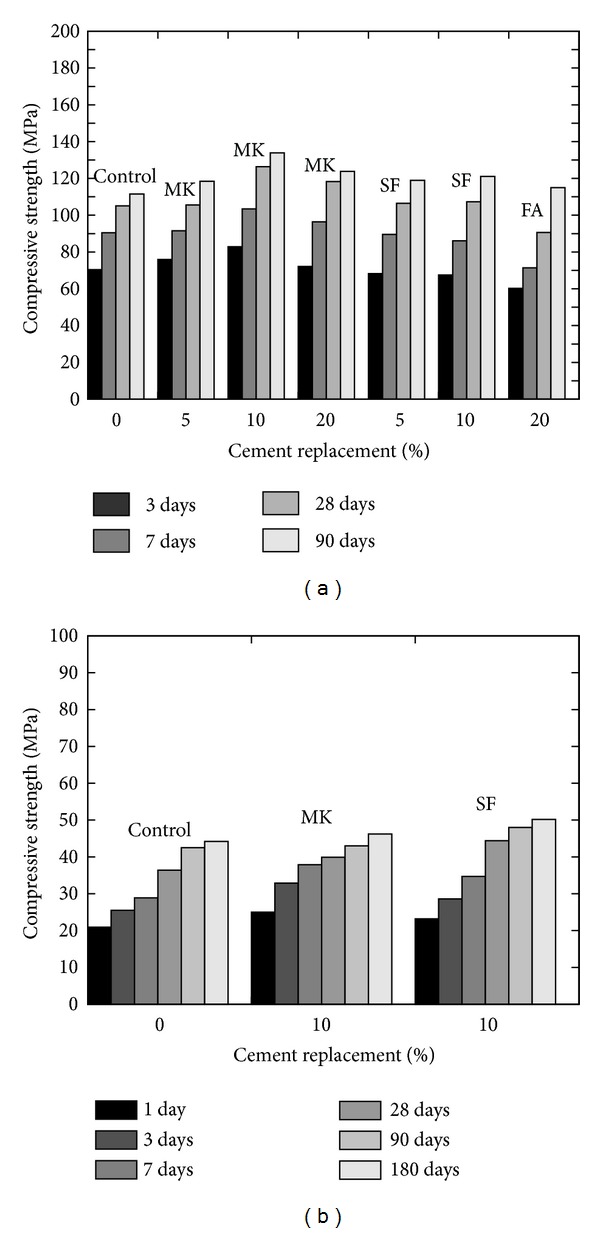
Effect of cement replacement on the compressive strength of (a) MK pastes compared to SF and FA pastes (w/b = 0.3) [12] and (b) MK concrete compared to SF concrete (w/b = 0.4) [30].

Poon et al. [12] achieved the best performance of cement pastes in terms of compressive strength at young age using 10% MK content as shown in Figure 11(a), but the use of high content of MK requires higher dosages of plasticizer [38]. The early strength gained is higher with the addition of MK in comparison with FA and SF [12]. Zhang and Malhotra [30] reported lesser compressive strength gain after 28 days; however they confirmed the faster rate of strength development in MK concrete than concrete at young age (refer to Figure 11(b)).

**Figure 12:**
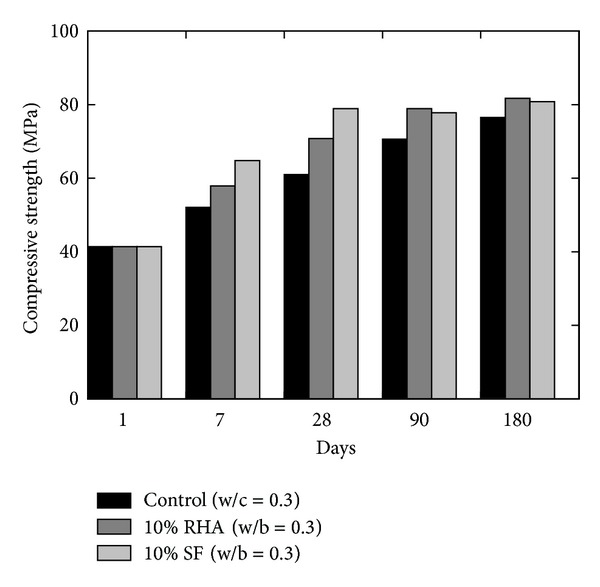
Performance comparison of high strength RHA and SF [21].

**Figure 13:**
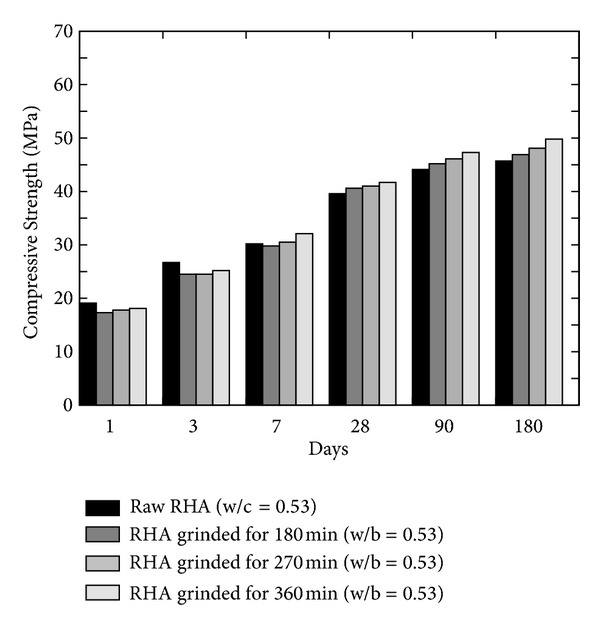
Effect of grinding timings on the compressive strength of RHA concrete [33].

**Figure 14:**
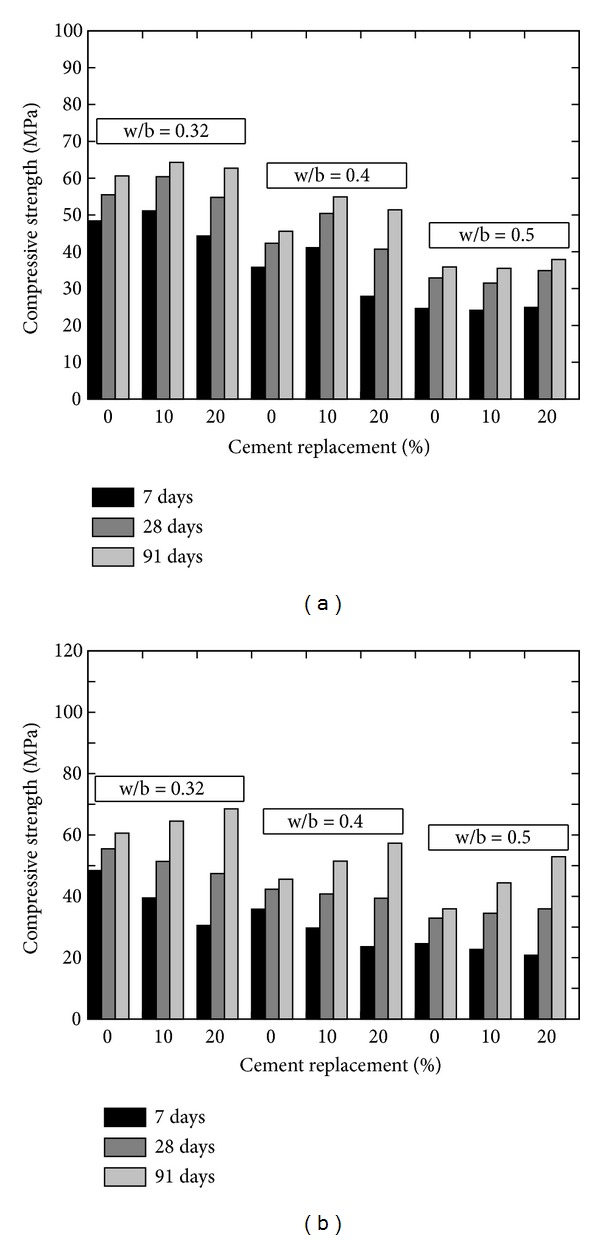
Effect of RHA imported from (a) USA (on left) and (b) Uruguay (on right) on the compressive strength [40].

**Figure 15:**
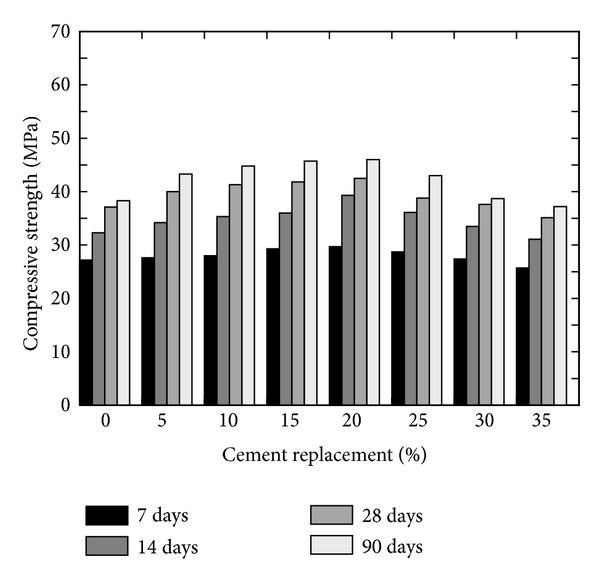
Effect of volume of cement replacement on the compressive strength of RHA concrete (w/b = 0.53) [5].

**Figure 16:**
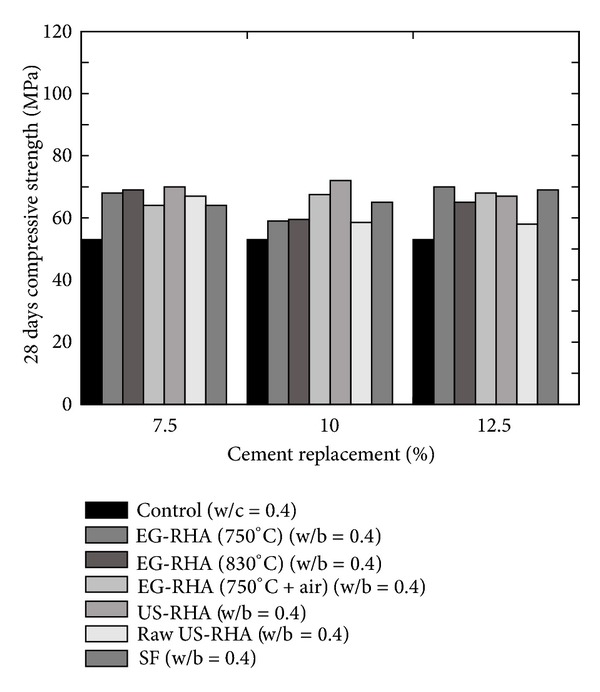
Effect of Egyptian RHA (EG-RHA) containing low carbon obtained at a different combustion temperature on the compressive strength of concrete [13].

Givi et al. [6] reported the optimum amount of cement that can be replaced with RHA of 10 to 30% in order to improve the compressive strength of RHA concrete and to achieve maximum long-term strength. Besides this, other researchers also suggested the contents of RHA by weight of the total cementitious material; for example, Mahmud et al. [39] reported the results of high strength concrete with 10% of RHA giving 80 MPa compressive strength at 28 days which is higher than concrete without RHA; however this strength was about 6% lower than the concrete in which cement was replaced by condensed SF. Similarly, Zhang et al. [21] reported (a) higher compressive strength with reduced porosity, (b) reduced calcium hydroxide content, and (c) reduced width of the interfacial zone between the paste and the aggregate (refer to Figure 12). Effect of fineness of RHA on the compressive strength was also analysed by Habeeb and Fayyadh [33] who reported that finer RHA shows higher strength of the concrete as compared to the coarser RHA (refer to the results plotted in Figure 13) due to the fact that finer RHA reacts more with Ca(OH)_2_ resulting in higher production of calcium silicate hydrate (C–S–H). Positive influence on the compressive strength at a young age was observed by Rodríguez de Sensale [40] who analysed the compressive strength development up to 91 days by replacing 10 and 20% of cement with RHA as shown in Figure 14. According to Rodríguez de Sensale [40], increase in compressive strength of RHA concrete is more significant at later ages. Similar results had been obtained by Alvarez [41] who described that the early strength of RHA concrete is lower than normal concrete; however the strength at 28 days is higher. Ganesan et al. [5] presented the results of concrete in which 15% cement content was replaced by RHA and they suggested that this replacement is optimum to obtain highest compressive strength (refer to Figure 15). On the other hand, El-Dakroury and Gasser [22] presented that 30% replacement of cement with RHA may be considered as optimum for all w/b ratios for mortars. Thus, it may be said that between 10 to 30% replacement of cement with RHA can provide a positive effect on the compressive strength of concrete. Nehdi et al. [13] reported a higher compressive strength of RHA concrete as compared to SF concrete having similar proportion (refer to Figure 16). Rice husk ash is a highly reactive pozzolan and produces high strength concrete; however RHA concrete requires relatively higher quantity of superplasticizer than OPC and SF concrete [34].

### 2.4. Splitting Tensile Strength

**Figure 17:**
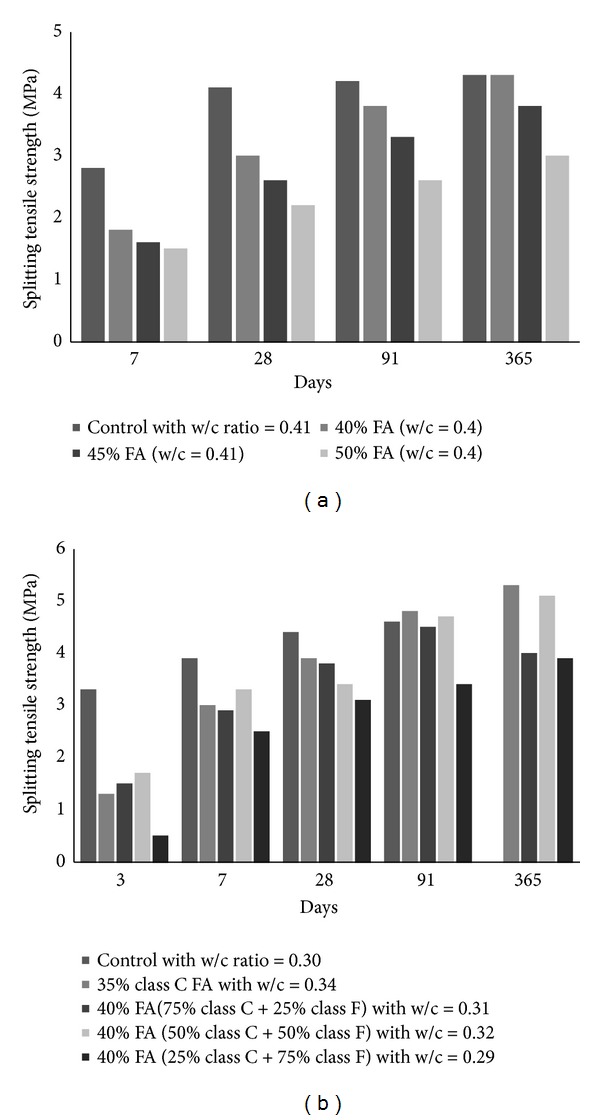
Effect of (a) FA type [36] (on left) and (b) cement replacement [26] (on right) on the splitting tensile strength of FA concrete.

Splitting tensile strength is the measure of tensile strength of the concrete which is determined by splitting the cylinder across its diameter. Siddique [36] reported a decrease in the splitting tensile strength of FA concrete when content of cement was replaced as 40, 45, and 50%; however, at all replacement level, compressive strength of FA concrete was higher than ordinary concrete. Naik et al. [26], on the other hand, presented the results of tensile strength of FA concrete in which class C and class F types were used together in varying proportions. The comparison of both studies has been shown in Figure 17. According to Figure 17, irrespective of the type and proportioning of FA and content of cement replacement, splitting strength of concrete increases with time, but not more than OPC at a young age. Therefore, it may be assumed that the addition of different types of fly ashes in different variations negatively influences the splitting tensile strength of concrete; however, replacement of fine aggregate by FA increases the tensile strength of concrete at replacement levels from 10–50% at all ages. The rate of increase in tensile strength with age decreases with the increase in replacement of cement with FA [3].

The tensile strength of GGBS concrete is reported to be slightly higher than that of ordinary Portland concrete [27]. Whereas, SF significantly increases the tensile strength of concrete, but it depends on the young age curing procedures and relative increase in compressive strength [27]. According to Zhang and Malhotra [34], the splitting tensile strength of high strength concrete incorporating SF is equivalent to high performance concrete, but lesser than RHA concrete at cement replacement level of 10% and water binder ratio of 0.4.

Splitting tensile strength of MK concrete is increased with the increase in MK content as cement replacing material. This has been confirmed by Qian and Li [38] who used MK from 5, 10, and 15% and reported an increment in average tensile strength as 7%, 16%, and 28%, respectively. Similar results have been reported by Justice et al. [11].

According to Zhang and Malhotra [34], the splitting tensile strength of high strength concrete incorporating RHA is comparable to high performance plain concrete; however Alvarez [41] observed that 28-day splitting tensile strength with 10% RHA is higher than normal concrete. Ganesan et al. [5] reported the 28-day splitting tensile strength of concrete with 0 to 35% of RHA with 5% variation. According to them [5], the splitting tensile strength increases as the RHA content increases up to 20% and, then at 30% RHA, the splitting tensile strength is equivalent to that of OPC concrete. Habeeb and Fayyadh [33] also reported that later age tensile strength of RHA concrete at 90 and 180 days is higher in comparison to concrete without RHA.

### 2.5. Flexural/Bending Strength

**Figure 18:**
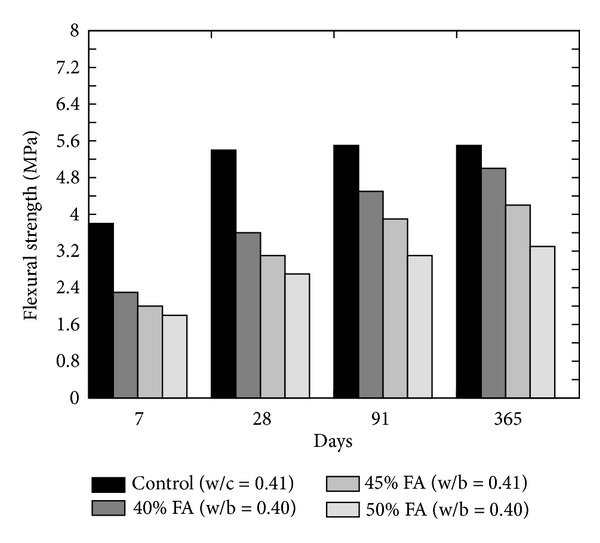
Effect of increasing FA content on the flexural strength [36].

Siddique [36] observed a decrease in the flexural strength as compared to the plain concrete at all ages when cement is replaced with 40, 45, and 50% of FA as shown in Figure 18. On the other hand, replacement of fine aggregate by FA increases the flexural strength of the concrete at replacement levels from 10 to 50% [3]. Flexural strength of concrete incorporating GGBS as the cement replacing material alone has not been found in the literature.

Flexural strength of SF concrete depends on compressive strength. An increase in compressive strength results in the increase in flexural strength. Moreover, it is also dependent on the young age curing method [27]. Zhang and Malhotra [34] reported that the flexural strength of high performance concrete containing SF is comparable to RHA at 10% level of cement replacement and constant water to binder ratio, but the flexural strength of both SF and RHA concrete was higher than that of the plain high strength concrete.

The increase in 28-day bending strength of concrete with 10 to 15% MK content as partial replacement of cement is reported as 32 and 38% whereas, at 80 days, bending strength of MK concrete is reported as 13 and 24%, respectively; however a small effect on the bending strength of MK concrete has been reported for replacement level of 5% [38]. Justice et al. [11] reported a higher flexural strength of MK concrete and SF concrete than ordinary concrete.

The addition of RHA does not cause a positive effect on the flexural strength of concrete at 28 days [41]; however the gain flexural strength is higher at 90 and 180 days in comparison to concrete without RHA [33]. Alvarez [41] reported that the flexural strength of concrete containing 10% RHA is higher than normal concrete.

### 2.6. Modulus of Elasticity

**Figure 19:**
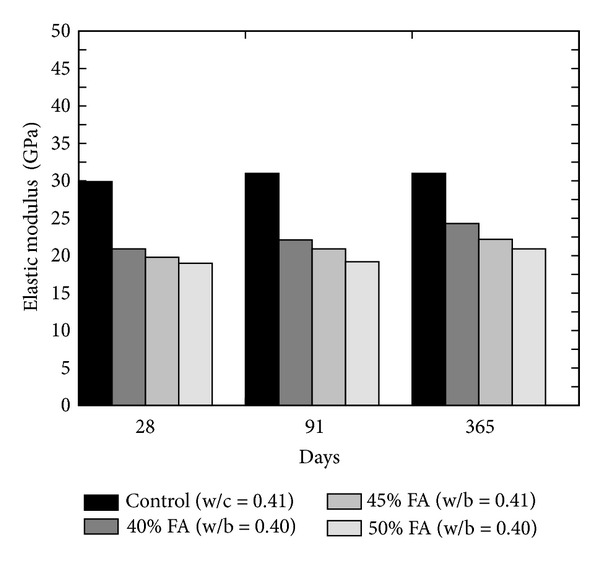
Effect of increasing FA content on the elastic modulus of concrete [36].

**Figure 20:**
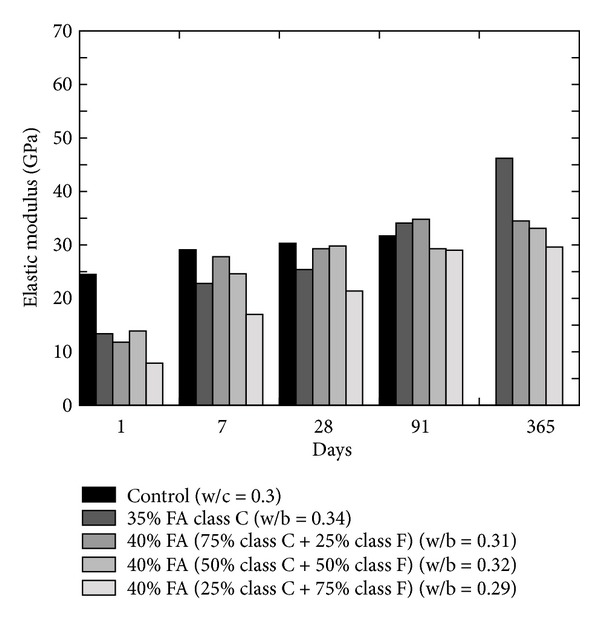
Effect of blended FA on the elastic modulus of concrete [26].

Modulus of elasticity is an important property to assess the resistance of concrete against freezing and thawing. This property can be determined by static as well as dynamic compression test. The literature reveals that the elastic modulus of FA concrete is generally equal to or slightly better than equivalent grade of concrete [27]. Ghosh and Timusk [23] reported that concrete containing good quality FA had equivalent modulus of elasticity value as that of normal concrete; however, decrease in modulus of elasticity was observed (refer to Figure 19) at the replacement level of 40, 45, and 50% [36]. Naik et al. [26] presented the effect of blended FA concrete containing class C and class F FA in different proportions as shown in Figure 20. At the age of 1 day, the elastic modulus results of OPC (i.e., control) concrete were the optimum, but, on later age, the difference in the elastic modulus of OPC and blended concrete reduced as the pozzolanic reactivity of FA increases with age; therefore, after 28 days, blended concrete showed higher results than the control.

Almusallam [25] determined the effect of w/b ratio on the initial and secant modulus of OPC and FA concrete and it can be seen from the results shown in the Table 3 that the modulus of elasticity increases with the increasing age and the difference in the initial and secant elastic modulus for the OPC is higher than FA concrete at w/c of 0.48 and 0.5.

In comparison to normal concrete, use of GGBS will slightly increase the elastic modulus for a given compressive strength [27]. The addition of SF in concrete does not follow the same trend in modulus of elasticity as in tensile strength and shows an insignificant increase [27]. The slight increase in elastic modulus has been shown in Table 4. Moreover, the reported results for HSC show that, at 10% SF, elastic modulus of SF concrete improves with decreasing water to cement ratio.

**Figure 21:**
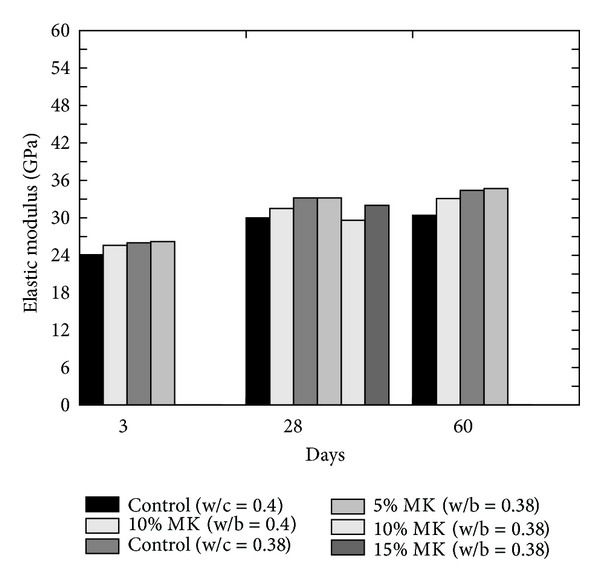
Effect of cement replacement on the elastic modulus of MK concrete [30, 38].

According to Zhang and Malhotra [30], the modulus of elasticity of MK concrete is higher than that of plain concrete and SF concrete for a w/c ratio of 0.38. Qian and Li [38] also confirmed it by reporting a small increase in the tensile and compressive modulus of elasticity of concrete with an increase in MK content using a w/c ratio of 0.4. The results of both studies [30, 38] have been shown in Figure 21.

According to Habeeb and Fayyadh [33], the static modulus of elasticity of concrete marginally increases by 20% RHA in concrete with higher dosage of superplasticizer (refer to Table 5). Zhang and Malhotra [30] showed comparable results of modulus of elasticity for concrete with and without RHA (refer to Table 5).

## 3. Conclusions

The purpose of this review was to have an idea about the role of different mineral admixtures on the mechanical characteristics of concrete because, in the designing of any structural element, these mechanical properties as well as the contribution of different mineral admixtures must be known. Prior to concluding this review, one should keep this fact in mind that these conclusions are general and purely based on the studies reported in this paper and the reported results may vary in different circumstances; for example, curing duration and period, casting methodology and workmanship, different particle sizes and different geographical source of mineral admixture and/or cement, and so forth may alter the properties of the concrete. Apart from this, the following conclusions are drawn.

1. Addition of mineral admixtures reduces pore size with increasing pore size distribution which in turn reduces porosity, permeability, shrinkage, and creep. The reduction of average pore diameter is highly and oppositely dependent on volume of cement replacement and age of concrete or paste; that is, with age and increasing volume of replacement of cement, average pore diameter and total porosity reduce.
2. Drying shrinkage and creep strains increase with the increasing age and volume of cement replacement and rate of increase in these strains is observed to be lower in pozzolanic concretes as compared to plain concrete.
3. Inclusion of mineral admixture improves the compressive strength of concrete. However, FA and GGBS are those mineral admixtures which enhance the later age compressive strength instead of early age due to their slow pozzolanic reactivity and/or lesser surface area. Since, in structural designing, 28-day compressive strength is important and use of these mineral admixtures may influence the design, therefore, based on the recommendations and quoted examples in the literature, combined use of slow and highly active mineral admixture can improve the performance of these mineral admixtures, such as MK and condensed SF.
4. Alongside compressive strength, addition of mineral admixtures slightly improves the tensile strength, flexural strength, and modulus of elasticity of concrete with the increase in replacement level. However, the case of FA is quite different from other mineral admixtures. The reported tensile and flexural strength of FA containing concrete is lesser due to slow compressive strength gain. The reported results of flexural and the splitting tensile strengths, modulus of elasticity, and drying shrinkage of the concrete incorporating RHA or SF showed that these two mineral admixtures are comparable. Therefore, any of the two can be chosen economically.

## Figures and Tables

**Table 1:** Average pore diameter of cement replaced hardened pastes.

Authors	Mineral admixture	w/c or w/b ratio	% content	Average pore diameter (nm)					Remark
				3 days	7 days	28 days	60 days	90 days	
Chindaprasirt et al. [15]	Control	0.35	0	—	—	23	19	15	Results estimated from Figure 2 of [15]. OFA had median particle size of 19.1 µm and CFA had 6.4 µm.
	Original fly ash (OFA)	0.35	20	—	—	22.5	18.75	13.75	
			40	—	—	20	17.5	13	
	Classified fly ash (CFA)	0.35	20	—	—	19	13.75	11.25	
			40	—	—	18	13	9.5	

Poon et al. [12]	Control	0.3	0	38	37.1	36.2	—	34.8	Results are borrowed from Table 3 of [12]
	Metakaolin	0.3	5	35.7	27.9	25.7	—	24.3	
			10	28.7	25.1	19.7	—	18.6	
			20	20.4	14.3	12.2	—	11.4	
	Silica fume	0.3	5	36.6	37	36.7	—	34.9	
			10	35.3	34.1	32.5	—	30.6	
	Fly ash	0.3	20	36.8	35.6	34.7	—	33.9	

Collins and Sanjayan [20]	Control	0.5	0	74.7	48.7	34.9	26.4*	—	Results are for pore radius, taken from Table 5 of [20]
	Alkali activated Slag	0.5	100	38.1	12.4	8.7	3.9*	—	

*Reported results are for 56 days.

**Table 2:** Total porosity of cement replaced hardened pastes after partially replacing the cement content.

Authors	Mineral admixture	w/c or w/b ratio	% content	Total porosity (%)					Remark
				3 days	7 days	28 days	60 days	90 days	
Chindaprasirt et al. [15]	Control		0	—	29.5	26.5	21.5	20	Results are approximated from Figure 2 of [15]
	Original fly ash		20	—	33	29	24	21.5	
		0.35	40	—	36.5	34	33.5	29	
	Classified fly ash		20	—	32	28.5	22.5	21	
			40	—	34	31.5	28	26.5	

Poon et al. [12]	Control		0	20.11	17.99	15.58	—	14.04	High performance cement pastes
	Metakaolin		5	18.17	15.36	13.82	—	12.51	
			10	16.84	15.18	12.37	—	11.68	
		0.3	20	16.3	12.85	10.73	—	9.21	
	Silica fume		5	18.72	16.83	14.53	—	13.84	
			10	16.97	15.49	14.23	—	13.42	
	Fly ash		20	22.35	18.59	15.62	—	13.82	

Collins and Sanjayan [20]	Control	0.5	0	34.7	34.4	35.6	33.6^¥^	—	Results are for pore radius, taken from Table 5 of [20]
	Alkali activated slag		100	33.2	32.2	31.4	24.3^¥^	—	

Bágel [10]	Control	0.52	—	—	—	—	—	10.39	Results are taken from Table 4 of [10]. Sand/binder ratio of 3 : 1 (by weight) was used and the amount of the water was varied to achieve a desired slump of 155 ± 5 mm
	50% S*, 0% SF	0.52	—	—	—	—	—	12.59	
	40% S*, 10% SF	0.56	—	—	—	—	—	14.68	
	30% S*, 20% SF	0.62	—	—	—	—	—	16.95	
	20% S*, 30% SF	0.68	—	—	—	—	—	21.22	
	10% S*, 40% SF	0.92	—	—	—	—	—	23.05	
	0% S*, 50% SF	1.06	—	—	—	—	—	27.75	
	50% S*, 5% SF	0.53	—	—	—	—	—	15.96	
	50% S*, 15% SF	0.60	—	—	—	—	—	20.96	
	50% S*, 25% SF	0.75	—	—	—	—	—	24.73	
	0% S*, 10% SF	0.56	—	—	—	—	—	15.28	
	0% S*, 30% SF	0.78	—	—	—	—	—	17.6	

El-Dakroury and Gasser [22]	Control		0	—	—	10.5	—	—	Results are approximated from Figure 7 of [22]
	RHA		10	—	—	8.25	—	—	
		0.5	20	—	—	5.75	—	—	
			30	—	—	4.5	—	—	
			40	—	—	5.5	—	—	
			50	—	—	6.75	—	—	

*S denotes slag.

^¥^Reported results are for 56 days.

**Table 3:** Elastic modulus of OPC and FA concrete [25].

Concrete type	Elastic modulus (GPa)	w/c or w/b ratio	Days			
			7	14	28	90
OPC	Initial		26.7	36.5	28.2	44.6
20% FA		0.48	29.3	27.5	26.4	38.8
OPC	Secant		23.9	28.9	26.4	35.1
20% FA			22.2	21.8	23.9	32.8

OPC	Initial		25.9	25.4	30.9	33.1
20% FA		0.5	23.2	22.7	25.3	38.1
OPC	Secant		21.9	22.5	26.6	28.9
20% FA			19.0	19.8	24.0	30.9

**Table 4:** Elastic modulus of SF concrete at 28 days.

Authors	Mineral admixture	% content	w/c or w/b ratio	Elastic modulus (GPa)	Remarks
Zhang and Malhotra [34]	Control	0	0.4	29.6	High strength concrete
	SF	10		31.1	

Mazloom et al. [29]	Control	0		34.4	Reported results are for the secant modulus of high strength concrete
	SF	6	0.35	35.5	
		10		37	
		15		38.1	

Sabir [2]	Control	0	0.5	33.5	Results of air entrained concrete are excluded
	Condensed SF	5	0.45	32.7	
		10	0.4	35.6	

Köksal et al. [42]	Control	0		33.8	Elastic modulus for HSC is taken from Table 3 of [42]
	SF	5	0.38	39.4	
		10		42.5	
		15		48.6	

**Table 5:** Elastic modulus of RHA concrete.

Authors	Mineral admixture	% content	w/c or w/b ratio	Elastic modulus (GPa)			Remark
				28 days	90 days	180 days	
Habeeb and Fayyadh [33]	Control	0		29.6	30.5	31	—
	RHA	20	0.53	30.1	30.8	31.4	Rice husk ground for 180 minutes
		20		30.2	31.4	31.7	Rice husk ground for 270 minutes
		20		30.5	32.3	32.9	Rice husk ground for 360 minutes

Zhang and Malhotra [30]	Control	0	0.4	29.6	—	—	High strength concrete
	RHA	10		29.6	—	—	
